# Hierarchical generative modeling for the design of multi-component systems

**DOI:** 10.1186/s13321-026-01313-3

**Published:** 2026-10-11

**Authors:** Rhyan Barrett, Robin Curth, Julia Westermayr

**Affiliations:** 1 Wilhelm Ostwald Institute for Physical and Theoretical Chemistry, Leipzig University, Linnéstraße 2, 04103 Leipzig, Germany; 2 Center for Scalable Data Analytics and Artificial Intelligence (ScaDS.AI), Dresden, Leipzig, Germany; 3 Present Address: Leibniz Institute of Surface Engineering (IOM), Permoserstraße 15, 04318 Leipzig, Germany

## Abstract

The functionality of catalysts, enzymes, and supramolecular assemblies emerges not from individual molecules alone, but from the subtle interplay between multiple components arranged in complex systems. Designing such systems is a grand challenge: the combinatorial explosion of possible chemical compositions and spatial arrangements makes brute-force exploration infeasible, while many current generative approaches remain limited to isolated molecules. In this work, we introduce a hierarchical generative optimization framework that overcomes this barrier by coupling a genetic algorithm for configurational search with a generative model for molecular design. This closed-loop approach enables simultaneous refinement of geometry and composition, efficiently steering discovery toward systems with targeted functionality. As a proof of concept, we design catalytic environments for the Claisen rearrangement of p-tolyl ether by optimizing surrounding components around a fixed reference transition-state geometry. Despite this constraint during the search phase, post-hoc validation via Climbing-Image Nudged Elastic Band calculations, with the environment held fixed, indicates a reduction of the activation barrier by about 30%. Beyond this example, the framework applies to systems in which a well-defined central structure is stabilized by non-covalently interacting components, such as supramolecular hosts and local catalytic environments.

**Supplementary Information:**

The online version contains supplementary material available at https://doi.org/10.1186/s13321-026-01313-3.

## Scientific contribution

The study presents a closed loop generative framework that enables joint optimization of molecular components and their spatial organization in multi-component systems. The method moves generative molecular design beyond single molecules toward larger and more complex systems.

**Supplementary Information:**

The online version contains supplementary material available at https://doi.org/10.1186/s13321-026-01313-3.

## Introduction

A central challenge in molecular discovery is the efficient exploration of chemical space to identify molecules and molecular systems with desired properties [1–3]. In cheminformatics, computational methods have become indispensable for this task by enabling virtual screening, property prediction, and data-driven prioritization of candidate structures before experimental testing [4–6]. However, the number of synthetically and functionally accessible compounds remains enormous, and structure property relationships are often highly non-linear, making exhaustive evaluation of predefined libraries increasingly impractical as molecular complexity grows [2, 7, 8]. As a result, many current workflows still rely on screening-based approaches that rank existing candidates rather than proposing fundamentally new ones [5, 6]. This limitation has motivated growing interest in inverse design strategies, particularly generative models that learn molecular representations from data and propose previously unseen structures tailored to target properties [9–11].

Generative machine learning models offer a powerful route to inverse design by learning statistical patterns in molecular data and proposing novel structures beyond those observed during training [12–14]. They can be trained on simple low-dimensional descriptors such as SMILES strings [15, 16] or on three-dimensional molecular structures. Prominent examples include autoregressive models such as G-SchNet [17, 18] and diffusion-based approaches [19, 20], which can generate molecular structures directly. When combined with supervised models that capture relationships between chemical structure and target properties, generative models can be steered toward desired regions of chemical space or conditioned to propose molecules with improved properties [18, 21–23].

Existing case studies, e.g., on the design of molecules using generative models for drug discovery and property optimization [22, 24, 25], demonstrate promising progress, but they remain largely restricted to isolated molecular systems. However, molecules rarely exist in isolation; instead, they function within complex chemical and biological environments. Examples include protein-ligand binding sites in enzymes, receptor ligand interactions in drug discovery, and solvation or molecular complex formation in condensed phases [26, 27]. In each of these cases, the functionality of the system emerges not only from the properties of the individual components but also from their intricate interactions [28]. In drug discovery, the environment is given and a single compound is optimized against it. Here we address the reverse problem, in which a central component is held fixed and the chemical identity of the surrounding components is the design variable. This setting arises in supramolecular host design [29] and in the design of local catalytic environments, the case treated in this work. The latter is related to the theozyme concept, in which model functional groups are arranged around a fixed transition state to quantify its stabilization [30]; here, however, the surrounding components are whole molecules generated de novo rather than motifs from a predefined catalogue, and their placement is searched jointly with their identity. Such systems are inherently multi-component, and because the design space of each constituent is already vast, the combined search space grows explosively when considered as a whole [31, 32].

Attempting to generate entire multi-component systems directly within a single generative framework proves extremely challenging, even when using conditional generative approaches. Three-dimensional generative models already degrade markedly with system size for single molecules: reported connectivity for EDM and GeoLDM falls from close to 99% on QM9 to below 50% on the larger GEOM-Drugs molecules [33], non-autoregressive models emit disconnected fragments more frequently as structures grow [34], and the cost of satisfying valence and connectivity constraints has been argued to grow exponentially with structure size [35]. A single-shot model for the systems considered here would have to satisfy these constraints for five molecules simultaneously while placing them in a mutually consistent, non-clashing relative geometry. When designing catalysts or reactive sites, not only the chemical composition needs to be optimized, but also the geometric features like relative orientation, distances, or symmetries of the individual components, to improve a reaction [36]. This combined compositional and configurational space is high-dimensional and heavily constrained by chemical validity and stability requirements. Consequently, existing strategies make the problem tractable by fixing one part of it in advance.

Deep learning approaches typically fix the chemical composition. Co-folding models such as AlphaFold3 [37] predict the structure of an assembly whose components are specified beforehand. Design pipelines split the problem into stages: RFdiffusion scaffolds a predefined motif geometry [38], ProteinMPNN and LigandMPNN assign sequences to a fixed backbone [39, 40], and PocketGen co-designs binding-site residues within a given scaffold [41, 42]. In each case the chemical building blocks are given and the model resolves structure or sequence. Classical hierarchical or multiscale workflows instead fix the candidate pool, first selecting a set of components and then optimizing their spatial arrangement using heuristic search strategies such as Monte Carlo sampling [43], evolutionary algorithms [44], or fragment-based assembly [45]. While effective in narrowing down large candidate spaces, these methods remain constrained by the diversity of the initial library and cannot autonomously propose new chemical components. In both settings the identity of the surrounding components is given rather than designed, leaving much of chemical space unexplored. A third line of work designs identity generatively, but for the catalyst itself. Ferraz-Caetano et al. generate vanadyl ligands de novo for epoxidation, co-designing ligand, substrate and reaction conditions [46]. Such approaches target the first coordination sphere, whereas here the reacting centre is fixed and the design variables are the identity and placement of the non-covalent components around it.

**Fig. 1:**
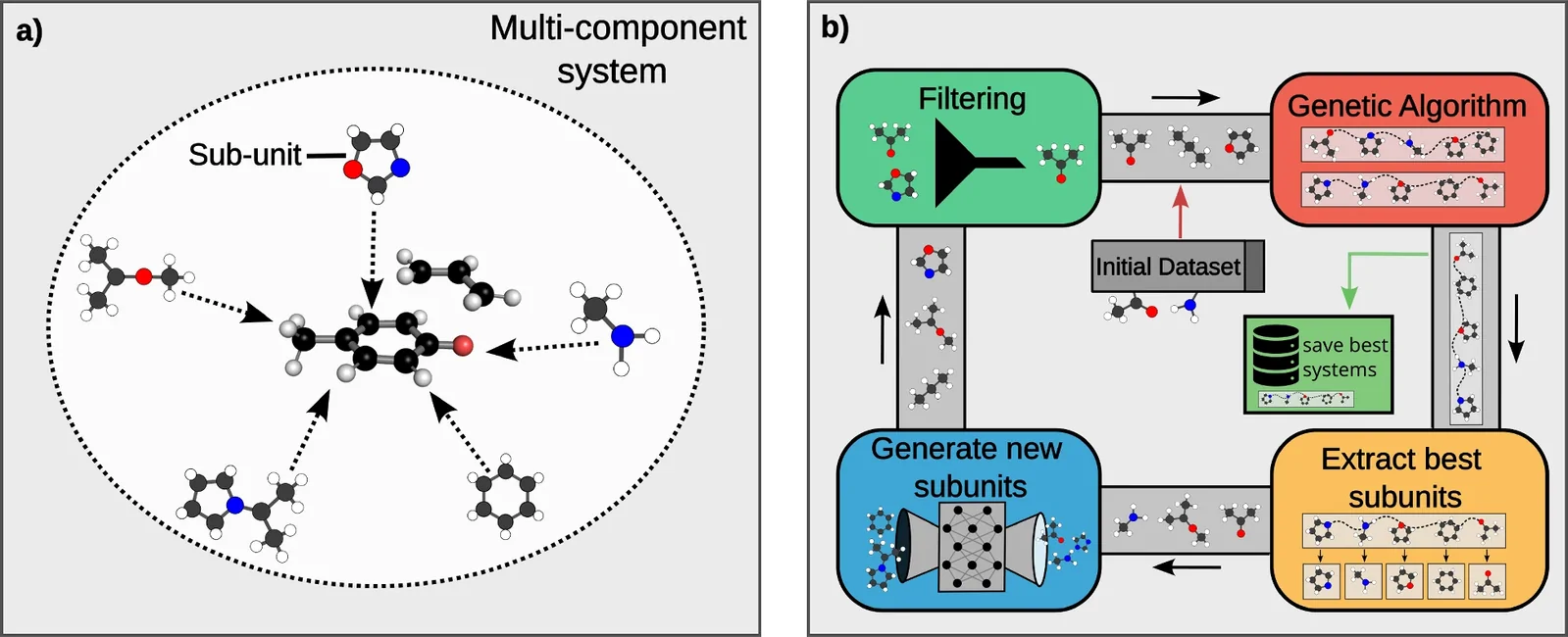
Overview of the developed multi-component structure design workflow. Panel a) illustrates the concept of a multi-component system, which is composed of molecular subunits in a specific spatial arrangement. Panel b) shows the developed multi-component design workflow. Starting with an initial dataset of molecules, a random population of multi-component-systems is constructed. The systems are handed to a genetic algorithm that optimizes the systems with respect to a target property. The molecules from the best-performing environments are collected and used as training data for a generative model that generates a new set of possible molecules to form the environment for each position. The loop is repeated until convergence of optimization the criterion

In this work, we combine generative design of chemical identity with explicit optimization of spatial arrangement in a single framework. Building on generative models that go beyond known chemical structures [47] and on hierarchical approaches to multi-component design, we propose a hierarchical generative optimization framework that breaks the problem into manageable sub-problems, as illustrated in Fig. 1. In the first stage, a global optimization layer explores the spatial arrangement of components by treating positions, distances, and orientations as tunable parameters. A genetic algorithm searches this configurational space to identify arrangements that minimize a chosen target property. Subsequently, a generative modeling layer focuses on the chemical composition of the subunits themselves. By learning from the best-performing environments found in previous iterations, the generative model proposes new molecules with similar or improved stabilizing features, enriching the pool of candidates for subsequent optimization. Together, these two stages form a closed-loop pipeline that iteratively refines both the chemical composition of the subunits and the global structure of the whole system. A key application of our framework is the automated design of local catalytic environments, where subtle changes in electrostatics, dispersion, or hydrogen-bonding can dramatically alter reaction rates. As a proof of concept, we apply the method to the Claisen rearrangement reaction of p-tolyl ether [48] and reduce the activation barrier by over 30%, revealing clear interaction motifs that stabilize the transition state, providing further chemical insights into design principles.

## Results

**Fig. 2:**
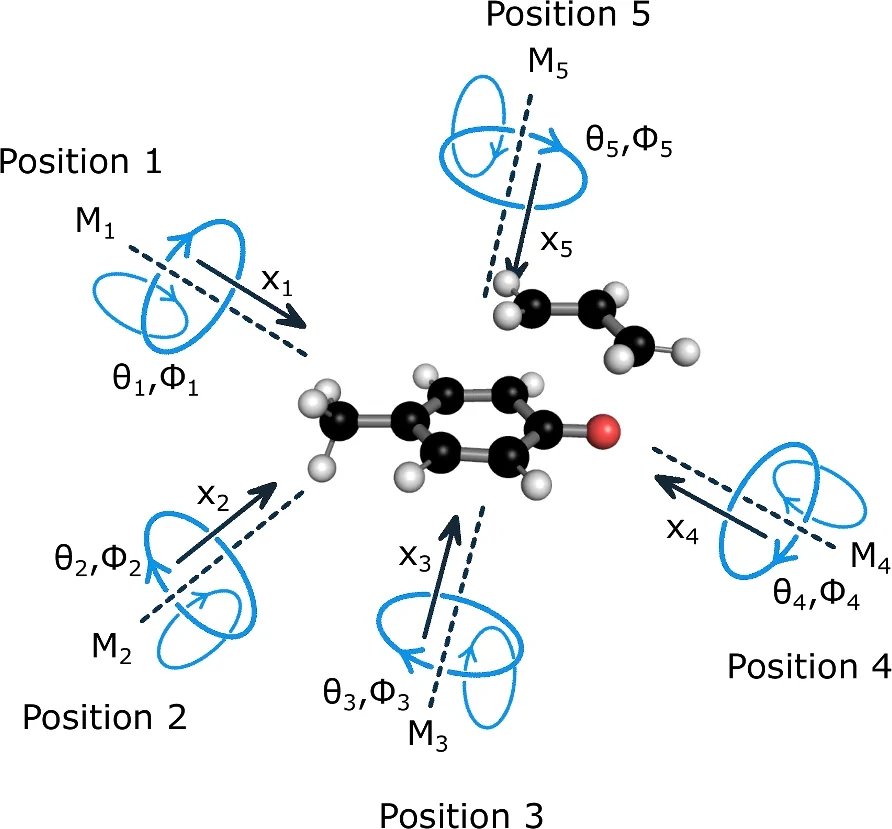
Transition-state test system and environment parameterization. The transition-state geometry is shown together with five predefined placement directions (positions j=1,⋯,5) for the surrounding molecular subunits. One subunit $M_j$ is assigned to each position *j* and is parameterized by $(x_j,\theta _j,\phi _j)$, corresponding to its distance from the transition state and two orientation angles that specify its placement and orientation

**Fig. 3:**
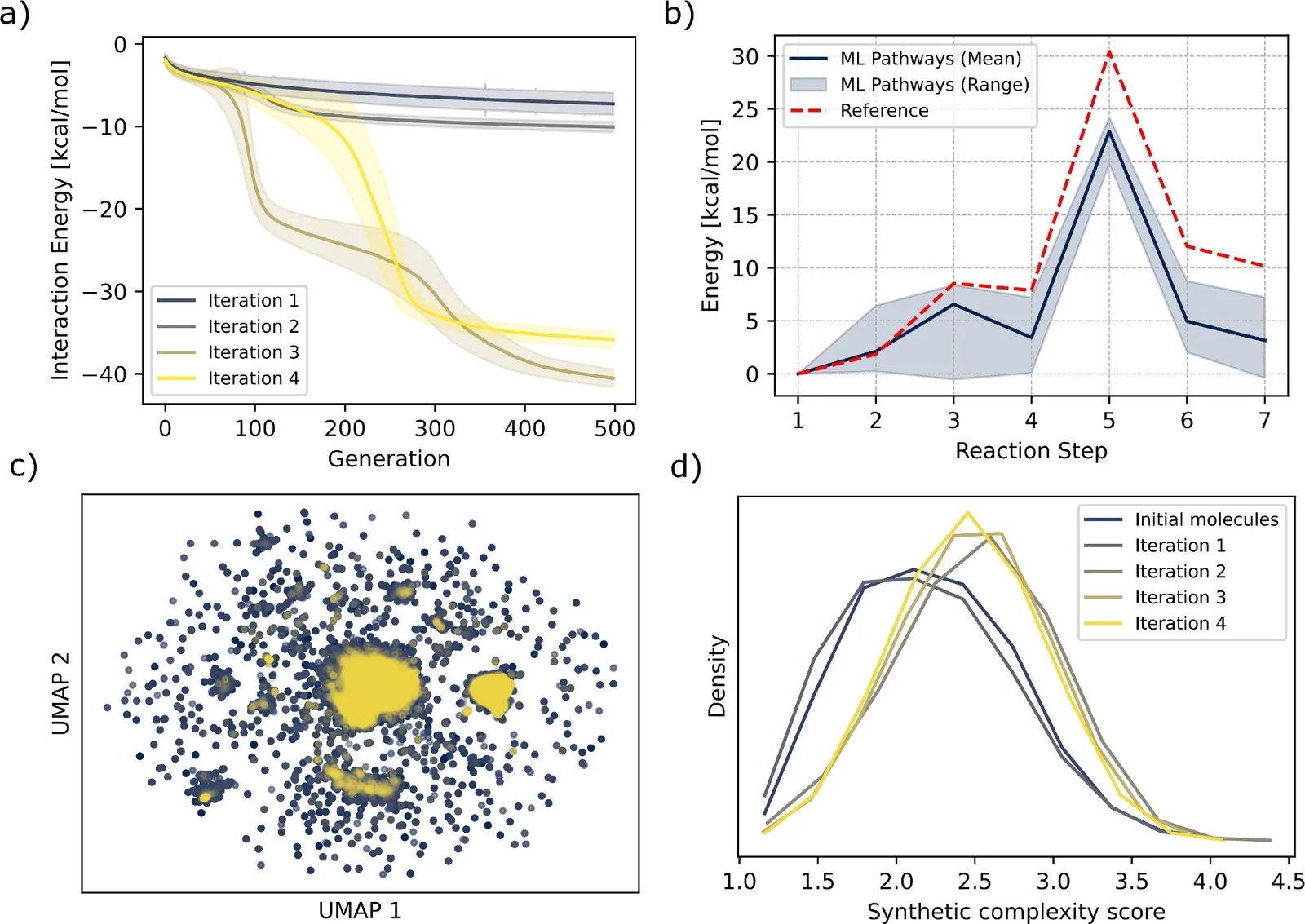
**a** Progress of the genetic algorithms. Solid lines represent the mean interaction energy of all generated local chemical environments with respect to the reference transition state and shaded areas show the standard deviation. **b** Reaction energy profile of the non-catalyzed reaction in vacuum (red) and of the reaction in 40 designed environments using NEB calculations. The blue line depicts the mean value obtained from all local environments, and the shaded area the standard deviation. **c** UMAP-projection of SOAP descriptors of the environment molecules, colored by iteration (the same legend as in c) applies). **d** Synthetic complexity score distributions of the environment molecules colored by generative iteration

**Fig. 4:**
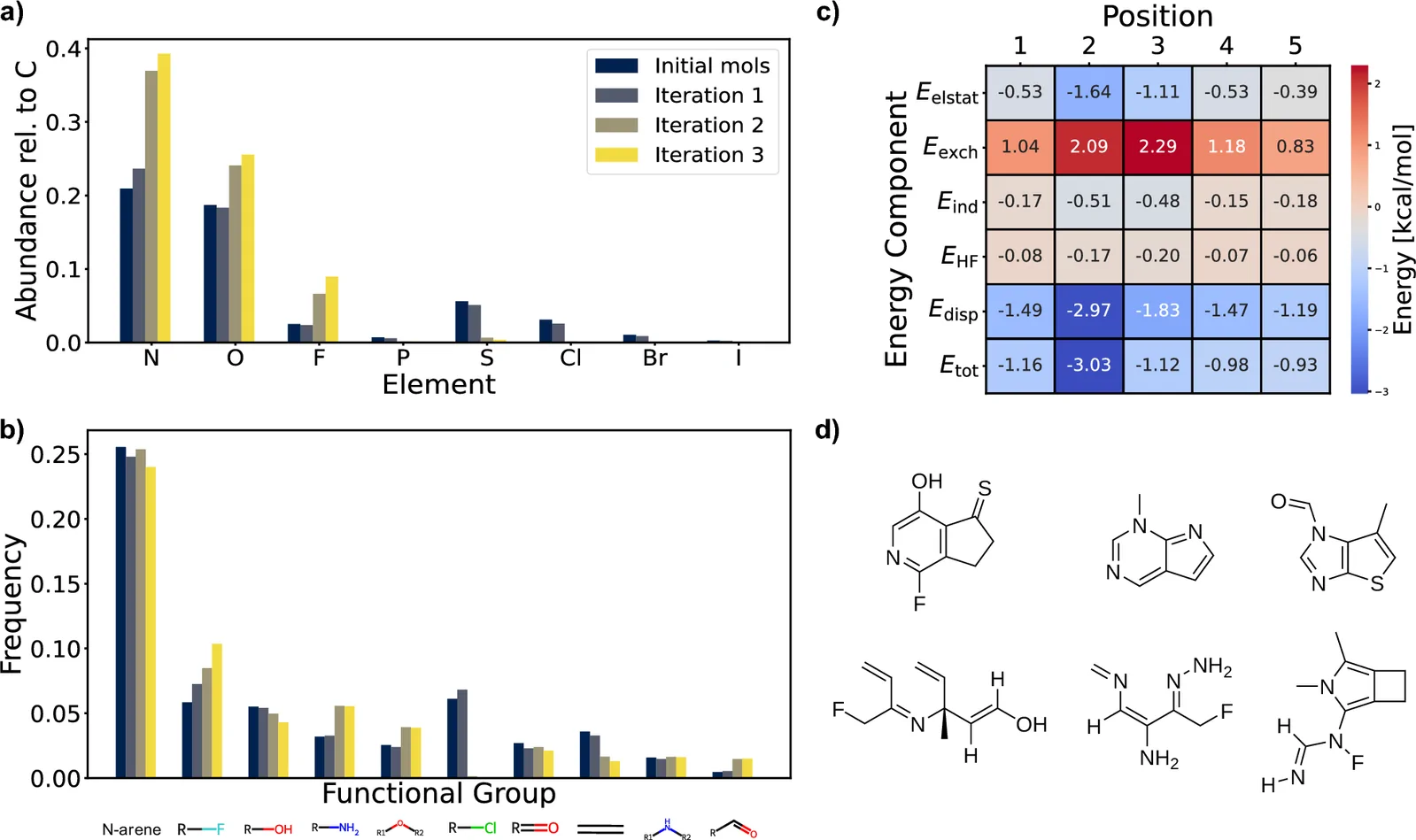
Development of **a** the atom type distribution and **b** the functional group distribution of the sets of environment molecules during the generative biasing steps. **c** Average SAPT interaction energy components between the environment molecules of the 40 environments chosen for further analysis and the transition state for each of the five environment vectors. **d** Selection of environment molecules from the last iteration

In this work, we apply the hierarchical multi-component optimization framework to the design of local chemical environments for reaction optimization by using the p-tolyl ether Claisen rearrangement reaction as an example. The proposed workflow is illustrated in Fig. 1 and will be explained in the following (Figs. 2, 3, 4).

### Multi-component system optimization workflow

**Fig. 5:**
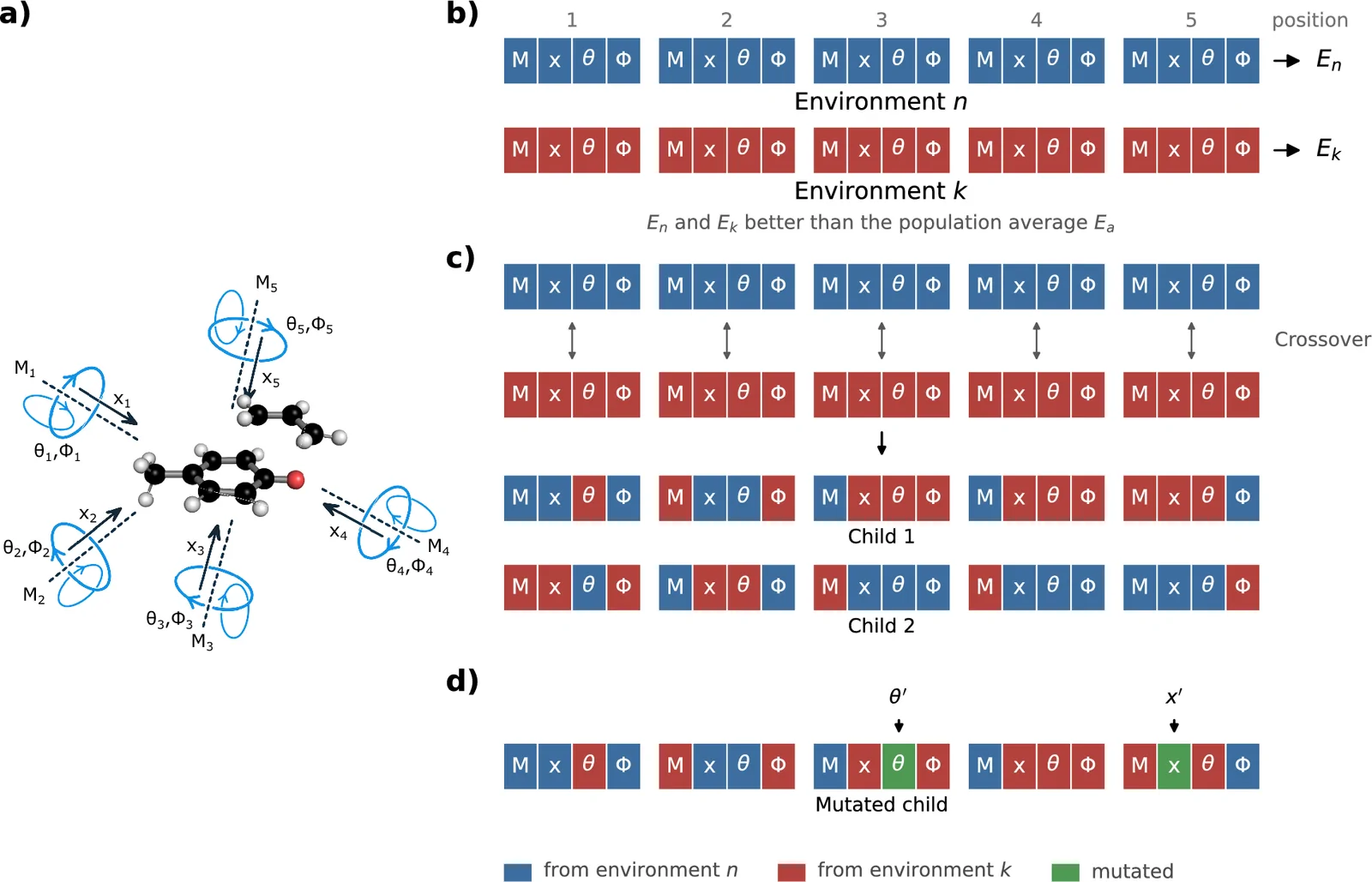
**a** Transition state of the Claisen rearrangement of p-tolyl ether with surrounding vectors characterized by four parameters: molecule index, intermolecular distance, and two angular coordinates describing the orientation of the molecules making up the local chemical environment. **b** Each environment, defined by its parameters, is evaluated through an energy prediction; environments performing above average advance to the next stage of the genetic algorithm. **c** New environments are generated from parent environments by randomly selecting and exchanging parameters. **d** With a certain probability, random parameter mutations are introduced into each environment

The developed workflow (Fig. 1) for designing a multi-component system can be understood as an assembly of several molecular or structural units placed in proximity to one another, as shown in the middle of the left side of Fig. 1. Each configuration is defined by two key elements: (i) a set of candidate building blocks that can occupy the system, and (ii) a global geometry that specifies how these blocks are arranged. The geometry is described by parameters such as distances, orientations, and positions, which together determine how the units are spatially organized, see also Fig. 5a. These two elements give rise to two coupled optimization problems: a chemical optimization problem, concerned with the identity and structural motifs of the building blocks, and a geometrical optimization problem, concerned with their spatial arrangement.

The geometrical optimization problem is addressed using a global optimizer, which we chose to be a genetic algorithm (GA) in this work (illustrated on the right of Fig. 1). At this stage, the pool of candidate molecular units is fixed, and the GA operates only on the parameters defining the overall structure. The GA requires three ingredients: a pool of molecular units, a predictive model for the target property, and the geometrical parameters that describe the arrangement. The optimization proceeds in cycles, illustrated in Fig. 1 (right side), referred to as generations, each of which follows four main steps: construction, selection, recombination, and mutation. In the construction step, which is shown in the orange box of Fig. 1 an initial population of structures is generated by randomly assigning molecular units to geometrical parameters, and each structure is scored, as illustrated in the red box on the right side of Fig. 1. Scoring is done in this work using a predictive machine learning model trained on quantum chemical properties of organic molecules (see MACE details section for details), but in principle, any scoring function can be applied that gives access to target properties.

In practice, as the optimization becomes more exploitative in later generations, the search can enter regions that are poorly represented in the training distribution of the underlying predictive model, leading to chemically implausible proposals. To prevent these from biasing the optimization, all candidates are subjected to chemical validity checks and structural relaxation prior to scoring, see the methods section for details. As foundational models improve in coverage and physical fidelity, we expect such failure modes to become less frequent, increasing the stability of coupled generative optimization workflows.

During selection, which is shown in the box on the bottom right of Fig. 1 the best-performing structures with respect to the pre-defined target property are retained, while recombination mixes their parameters to generate new candidates. Mutation (shown in the light-red box of Fig. 1) then introduces small random variations to maintain diversity and avoid premature convergence. Repeating this cycle over many generations allows the GA to refine the global arrangement, propagating favorable configurations while still exploring new possibilities. The full details of the GA can be found in the methods section and Fig. 5.

After the geometrical optimization with the GA converges, the molecular units that most frequently appear in high-performing systems are collected and used to retrain a generative model that was previously trained on an initial pool of potential candidates (see left part of Fig. 1). By biasing towards promising systems, this model proposes new candidate units with features similar to those found in the previous best-performing structures. A new round of geometrical optimization is performed with the new pool of candidates. By alternating between genetic optimization of geometry and generative modeling of building blocks, the workflow systematically improves both the arrangement and composition of the system until the target property reaches convergence. In principle, one could replace this two-stage loop with a fully generative end-to-end approach that directly proposes complete multi-component assemblies. However, maintaining chemical validity and stability of such large assemblies after construction is substantially more challenging than for small molecules; we therefore use the genetic algorithm as a search layer and the generative model to produce smaller, more stable molecules.

### Case study: application to local catalytic environments

Building on the general workflow described above, we applied the approach to the design of a local catalytic environment for the Claisen rearrangement of p-tolyl ether. The environment was represented by five molecular subunits placed along predefined vectors surrounding the transition state, as can be seen in Fig. 2. At initialization, one subunit is assigned to each of the five positions by random sampling from the SPICE dataset, which provides the candidate pool used throughout the workflow. Initially, for each vector *j*, the subunit distance $x_j$ is drawn uniformly from a feasible range and its orientation is randomized via the angles $(\theta _j,\phi _j)$; the GA then optimizes both molecular identity and these placement parameters. Five placement vectors were chosen as an arrangement that permits several simultaneous non-covalent contacts with the reacting complex. The number of subunits is a free parameter of the workflow and can be changed by adding or removing placement vectors. Each vector was parameterized by one distance and two angular degrees of freedom, defining how a molecule can be positioned and oriented relative to the reaction center. A pool of candidate molecules was assigned to each vector, from which the GA constructed and optimized local environments.

Although we consider five positions around a single transition state here (Fig. 2), the framework is not specific to this reaction; the conditions under which it is expected to apply are set out in the Discussion. For other applications, the number and placement of positions would be chosen to match the underlying scaffold structure, while the pool of molecular subunits would be selected to reflect the domain of interest. In a prospective application to a protein binding site, for example, positions could be defined by pocket anchor points and candidate subunits could be amino-acid variants at these anchors, with scoring based on predicted binding affinity. No such application is attempted here; all subunits in the present work are small organic molecules.

In this example we define the target property to be the interaction energy between the vacuum transition state and the environment, defined as

1 $$\begin{aligned} \delta E = E_{TS_{env}} - (E_{env} + E_{TS}), \end{aligned}$$

where $E_{TS_{env}}$ is the energy of the full system, and $E_{env}$ and $E_{TS}$ correspond to the isolated fragments, i.e., the environment and the transition state of the target reaction, respectively. We emphasize that δE is a screening objective and not the activation barrier. It is evaluated for a fixed gas-phase transition-state geometry and measures only the interaction with the transition state, whereas the kinetic barrier is determined by the relative stabilization of the reactant minimum and the barrier region along a relaxed minimum-energy path. An environment that stabilizes the transition state may therefore also stabilize the reactant or an intermediate, so a favorable δE does not by itself guarantee a lower barrier. For this reason δE is used only to rank candidates during the search, and the reported barriers are obtained separately from climbing-image Nudged Elastic Band (NEB) calculations at the DFT level. To evaluate δE for the large number of candidates generated during the search, we use the message-passing atomic cluster expansion model (MACE), in particular the MACE-OFF23 model [49], which is trained on millions of organic molecules. This model is finetuned on reference calculations of randomly generated environments computed at the ωB97M-D3BJ level of theory [50, 51], where the environment subunits are sampled from the SPICE dataset [52] as a source of candidate fragments; the finetuning labels are newly computed DFT interaction energies for the constructed Claisen transition-state environment complexes to refine the predictions for our specific system. While we use the fine-tuned model in this work, the optimization loop can in principle also be run with a non fine-tuned foundational model. Further details regarding the reference calculations and the finetuning can be found in the supporting information (SI) in section S1.

After each optimization cycle, the subunits most frequently present in high-performing environments are collected to guide the generative stage. For this, SiMGen [47] is used. SiMGen is a generative extension of MACE [53], which proposes a new set of candidate molecules conditioned on top-performing structures. Using these new candidates, the genetic optimization is repeated. This alternating procedure of geometric optimization and generative expansion is performed until convergence is reached and no further improvement in δE is observed.

The most promising environments from the final iteration are then validated against quantum-chemical calculations and analyzed to provide chemical insights and potentially guide further design and optimization processes.

The results can be seen in Fig. 3. In total, four optimization runs were conducted until the interaction energy did not improve further. Here, four optimization runs refers to four outer-loop iterations of the hierarchical workflow, where each iteration consists of a GA run of 500 generations followed by a generative update of the subunit pool. We stopped the outer loop once the mean predicted interaction energy no longer improved compared to the previous iteration (Fig. 3a); an additional iteration did not yield further gains in the ML score. Panel a) shows the mean predicted interaction energy as a function of generations of the GA for each run. As can be seen, each individual run of the GA shows a downward trend in the target property and convergence in the late stages of the GA. While iteration 1 converges towards a value of approximately −7.4 kcal/mol, iteration 2 reaches a value of approximately −10.2 kcal/mol. This improvement can be attributed to the generative biasing step, which shifts the distribution of candidate molecules toward those more potent at stabilizing the transition state. Iteration 3 converges at a much lower average interaction energy of about −40.6 kcal/mol, and the development of the interaction energy shows a stepwise decrease. Analysis of the molecules at each position shows that the stepwise decrease in interaction energy is caused by the algorithm discovering a highly optimal configuration, which results in extensive exploitation. Iteration 4 converges at around −35.7 kcal/mol, which is marginally higher than that of iteration 3. Further runs did not show improvement, and hence the process was stopped after this iteration (see SI Sec. S8 for the additional fifth iteration). Taken together, the iterations also function as an ablation of the two stages of the workflow: iteration 1 runs on the unbiased SPICE library and therefore isolates the contribution of the configurational search alone, while the generative stage is active only from iteration 2 onward. The improvement across the later iterations is attributable to the biasing of the candidate pool, since this is the only component that changes between them, although the magnitude of the gain at iteration 3 reflects a single run and should not be over-interpreted. The converse ablation, generative biasing without the genetic algorithm, is not well defined in this setup, as the biasing requires the scored top-performing environments produced by the search as its conditioning signal. To validate whether the modified local environments lead to an increased stabilization of the transition state, hence an increased reaction rate, we validate the reaction using the NEB method [54] at the Density Functional Theory (DFT) level to confirm the reduction in activation energy on a set of the best performing environments from the last iteration. Here, the reported approximate activation energy is defined as the energy difference between the reactant minimum and the highest-energy climbing image along the CI-NEB path, with the environment kept fixed, providing a consistent comparative barrier estimate across environments. The NEB method optimizes a reaction path and searches for the minimum energy pathway for each local environment. We have selected 40 top-performing local chemical environments for validation. The resulting energy curves from the minimum energy paths of the different designed local chemical environments can be seen in Fig. 3b. As can be seen, the activation energy is reduced from ∼30 kcal/mol to ∼20 kcal/mol on average when comparing the original (vacuum) pathway with those of the top 40 environments. This reduction should be interpreted within the constraints of the present computational setup. The surrounding components are kept fixed during the CI-NEB calculations, and the present proof-of-concept model does not include a larger structural scaffold that would constrain these components in a realizable catalytic system. The transition-state geometry itself, by contrast, is relaxed during these calculations, so the displacement of the transition state in response to the environment is captured rather than assumed to be absent; this displacement is quantified relative to the gas-phase reference in SI Sec. S9. The reported decrease therefore represents computational stabilization within a restricted local environment rather than the behavior of a complete, fully flexible catalytic system. These calculations validate the use of the finetuned MACE version to assess the propensity of local chemical environments to stabilize the transition state. To investigate how and why certain molecules are more likely to lead to an energy stabilization, we analyze the change in the distribution of environment molecules during the different iterations. Therefore, each position in the local environment is treated separately and molecules are represented using the Smooth Overlap of Atomic Positions (SOAP) [55] descriptor. SOAP embeds a molecular structure in a high-dimensional space, providing a way to mathematically represent the chemical structure space spanned by a set of molecules. A Uniform Manifold Approximation and Projection (UMAP) [56] dimensionality reduction was performed for visualization with details specified in the SI. UMAP takes the structural SOAP descriptor and transforms it to a lower-dimensional space, while preserving as much of the global structure of the data distribution as possible. Fig. 3c shows the distribution of the molecules in this new structure space spanned by the two UMAP components. It can be seen that the molecules become less diverse with increasing iteration and span a narrower chemical space. This finding is reasonable as certain molecular motifs are more likely to stabilize the transition state than others and are identified during the iterative cycles. Figure S4 in the SI shows the chemical space spanned for each position in the local chemical environment separately. To obtain an indication of the synthetic complexity of the generated molecules, we additionally plot the distribution of synthetic complexity scores (SCScore) [57], illustrated in Fig. 3d. A higher SCScore indicates more complex synthesis, smaller values are generally preferable. As a learned score, SCScore should be regarded as a measure of relative molecular complexity rather than a retrosynthetically accurate measure of true synthetic accessibility, but it remains a well-established and computationally inexpensive metric for comparing trends across datasets. A further caveat is that SCScore was trained on a corpus of literature reactions, so its reliability decreases for molecules lying outside this distribution. As the generated subunits drift away from the initial dataset over the iterations (Fig. 3c), the values reported here should be read as the direction of the complexity shift rather than as calibrated estimates for individual molecules. As can be seen, after the first iteration, the distribution is almost identical to the original data set but shifts slightly to larger values in iteration 2, where it remains nearly constant in further iterations. Notably, some of the highest performing candidates as shown in Fig. 4d likely fall into the upper tail of the SCScore distribution, indicating a trade-off between predicted catalytic stabilization and synthetic accessibility. This suggests that while the model can identify strongly stabilizing environments, a subset of these designs may be challenging to realize experimentally. In future works, this can be addressed by complementing SCScore with retrosynthesis predictions (e.g., predicted route length or feasibility) and incorporating these as additional filters or as a secondary objective during generative biasing.

This shift in predicted synthetic complexity is likely caused by the transition from known literature-based molecules towards machine learning-generated molecules, which may compromise the synthesizability as a side result. Still, given the general much lower synthesizability and plausibility of molecular structures generated with generative machine learning in recent studies, the SCScore can be considered fairly low [11, 21]. This result is further supported by distribution of bond lengths and angles plotted in Figure S3 in the SI comparing generated and original molecular structures.

#### Chemical insights and design principles

Finally, to gain a better understanding of what influences the reaction rate, chemical analysis of the molecules that lead to a stabilized transition state is performed. Rather than interrogating the surrogate model, as is common in explainable AI approaches to molecular design [58], we characterise the resulting environments directly, so that the reported motifs are established independently of the model used to select them. Therefore, after each generative phase converged, molecules that lead to the highest stabilization energy increase are collected. As these molecules are also used to bias the generative model, the elemental composition of the environment molecules changed significantly with each iteration and generation step, which is shown in Fig. 4a. The proportion of fluorine, nitrogen, and oxygen relative to carbon increases over the course of the iterations, while the occurrence of larger halogens (Cl, Br, I) and third-row heteroatoms (P, S) decreases. No explicit constraints or penalties were applied to these elements; instead, this shift emerges from the iterative selection-and-biasing loop, where the GA preferentially retains environments with favorable predicted interaction energies and the generative stage is subsequently conditioned on the subunits enriched among these top-performing environments. The enrichment of the more electronegative elements F, N, and O is consistent with a possible increased role of electrostatic interactions between the transition state and its environment. However, this compositional trend alone does not establish such a relationship quantitatively, and we therefore regard this interpretation as a hypothesis motivated by the observed enrichment. The nature of the non-covalent interactions in the final environments is examined separately below using SAPT. To further monitor changes in the molecules that make up the local chemical environment of the reaction, different functional groups were investigated. These were selected based on structural motifs that were observed via visual inspection of molecules that led to improved stabilization energy. The development of the frequency of the ten most common functional groups found in the datasets is shown in Fig. 4b. The most common functional groups are N-heterocyclic aromatic groups, followed by fluorinated groups, alcohols, and primary amines. As already discussed, the proportion of fluorinated groups increases, and the proportion of chlorine drastically decreases. Primary amine groups, ether groups, and aldehydes become more frequent, while carbon-carbon double bonds become less frequent. However, apart from the enrichment in fluorine and the decline in chlorine content, no specific functional group stands out as the one that is best-suited for catalyzing the reaction.

The environment is represented by vectors that constrain the positioning of molecules around the transition state and exhibit distinct behaviors during the genetic optimization cycles and the generative biasing stages. The relative orientation of the different vectors to the transition state might lead to different types of interactions, e.g. hydrogen bonding interactions occur if molecules are positioned close to the oxygen atom of the transition state. To gain insights into the nature of non-covalent interactions between the environment molecules and the transition state, and how they differ between the five different vectors that constrain the system, we performed Symmetry Adapted Perturbation Theory (SAPT) [59] calculations of the best-performing environment structures of the last iteration. SAPT decomposes the non-covalent interaction energy between two fragments into electrostatic, induction, dispersion, and exchange contributions.

The calculations were done in a pairwise fashion, with the transition state and one of the environment molecules as fragments at the PBE0/aug-cc-pVDZ [60] level of theory. The resulting average interaction energy contributions for each position are shown in Fig. 4c. The SAPT calculations show that the strongest average interactions occur between molecules at position 2 (Fig. 2), with total interaction energies about three times larger compared to the other positions. This strong attractive interaction at position 2 is driven by large dispersion interactions and electrostatic interactions, as well as a large induction component. Position 2 is located close to the aromatic ring of the transition state. Based on the energy profile of the interaction and the orientation of position 2, it is expected that strong interactions stem primarily from π-stacking interactions. The large proportion of N-heterocyclic aromatic molecules in the datasets supports this hypothesis. Panel c in Fig. 4 further shows that electrostatic interactions are strongest at positions 2 and 3, which can be explained by interactions with the electron-rich oxygen atom of the transition state, for example, via hydrogen bonding. The other environment vectors are positioned further away from the oxygen atoms (see Fig. 2), leading to stronger electrostatic interactions. Figure 4d displays selected molecules from the final iteration that are capable of engaging in pi-stacking interactions (top row) and hydrogen-bonding interactions (bottom row), both of which have been shown to favorably stabilize the transition state. The use of hydrogen-bond donors as catalysts for the Claisen rearrangement reaction has already been reported in the literature [61].

## Discussion

In this work, we develop a hierarchical generative framework for efficient, property-driven design of multi-component molecular systems. The approach decomposes the design task into two coupled but tractable subproblems: global spatial optimization via a genetic algorithm and local subunit generation via a molecular generative model. By alternating between these stages, the workflow can move beyond a fixed initial library while optimizing the spatial context in which molecules act. In the present proof-of-principle, the transition-state geometry is kept fixed during optimization, such that the framework identifies environments that stabilize a given reference structure and thereby isolates the effect of the surrounding multi-component arrangement on reaction energetics. In a prospective application, the optimized local components would additionally need to be embedded in a structural scaffold, such as a protein backbone, supramolecular host, or other molecular framework, that constrains their relative positions. Extending the approach to cases where the environment actively reshapes the reaction pathway will require coupling the search to iterative transition-state or reaction-path optimization, which represents a natural direction for future work.

Within these bounds, the workflow is not a universal design method, and the Claisen rearrangement case should be read as a demonstration rather than as evidence of general applicability. The approach requires three conditions. First, the system must contain a well-defined central structure, here the transition-state geometry, rigid enough to be held fixed while its surroundings are optimized, so that every candidate environment is scored against the same reference. Second, the target property must be a scalar objective dominated by non-covalent interactions with discrete surrounding components, cheap enough to evaluate for the many candidates visited by the genetic algorithm, and within the domain of the surrogate model, here neutral closed-shell organic molecules. Third, the components must be placeable along a small number of predefined directions, keeping the configurational search low-dimensional.

A suitable application is therefore a non-covalent host or co-catalyst environment around a rigid guest. The self-assembled Ga$_4$L$_6$ tetrahedron of Fiedler, Bergman and Raymond is one such case, its cavity holds an encapsulated guest in a reactive conformation and accelerates an aza-Cope rearrangement without bonding to the substrate [62], as do hydrogen-bond-donor catalysts of the Claisen rearrangement itself [61], which correspond to the motifs recovered in Sec. 2.2.1. An unsuitable application is a transition metal catalysed reaction in which the catalyst bonds covalently to the substrate, since the operative pathway then depends on the ligand, oxidation state and additives [63]: the reference geometry becomes a function of the environment, and δE no longer measures a fixed quantity. Systems with many low-lying conformers [64], or dominated by bulk solvent rather than discrete contacts [65], fall outside the present scope until the search is coupled to iterative reaction-path re-optimization.

To enable property-driven optimization, the workflow requires a scoring function, which we implement here as a predictive machine learning model. This dependence requires reliable supervised models, since inaccurate predictions can bias the search toward misleading candidates. This risk can be mitigated by employing ensemble models or explicit uncertainty quantification. By identifying structures with highly uncertain model predictions, targeted reference DFT calculations can be performed in an active-learning scheme, to update the model as the chemical distribution shifts during the iterations. We have shown that molecules generated in this work remain chemically valid, with complexity scores comparable to the initial dataset. Additional screening and biasing using predictive models for synthesizability or scoring functions therefore can further improve the generative stage. Uncertainty estimates are best used to prioritize which candidates to label with reference calculations rather than to certify individual predictions, since the estimates themselves can be unreliable outside the training distribution, typically underestimating the true error. The global optimizer is similarly interchangeable; we use a genetic algorithm because the objective is cheap to evaluate and because crossover and mutation act naturally on the mixed discrete and continuous parameters of each position, but any method that proposes candidate assignments and consumes a scalar objective could be substituted.

Compared to conventional approaches, which often rely on fragment-based assembly [45] or heuristic search [43, 44] without generative exploration, our method combines the strengths of both optimization and generation. While generative design in catalysis has so far largely targeted the catalyst itself [46], this framework extends the concept to complex environments such as catalytic pockets, enzyme active sites [30], and supramolecular assemblies [29], where interactions emerge from both chemical composition and spatial arrangement. We locate the main contribution of this work in this multi-component formulation, in which the identity and the placement of the surrounding components are optimized jointly rather than either being fixed in advance. The chemical motifs identified in Sec. 2.2.1, namely π-stacking at position 2, electrostatic and hydrogen-bonding contacts to the ether oxygen, and enrichment in F, N and O, are a corroborating result rather than a separate claim: hydrogen-bond donor catalysis of the Claisen rearrangement is already established [61], and the recovery of such motifs indicates that the optimization converges on chemically sensible interactions rather than artefacts of the scoring model.

Future work will aim to combine the approach with more robust machine learning potentials, improved neural network architectures, or explicit uncertainty modeling to enhance predictive accuracy. Recently developed foundational models [66, 67] provide avenues for improved predictions. In addition, expanding the framework to other use cases, such as the design of functional enzyme pockets or tailored supramolecular assemblies, will help test the applicability of the workflow in broader contexts.

## Methods

### Overview of the workflow components

The components described in this section play three distinct roles, with different error sources and different consequences if they fail.

The genetic algorithm, the MACE-OFF23 scoring model, and the SiMGen generative model constitute the optimization stage. They operate inside the closed loop and determine which environments are designed: the genetic algorithm searches the spatial arrangement, MACE-OFF23 scores each candidate through the interaction energy of Eq. (1), and SiMGen biases the candidate pool between iterations. Errors in these components propagate into the design itself, through surrogate error against the ωB97M-D3BJ reference, through the stochasticity of the search, and through invalid or out-of-distribution generated structures, respectively.

The CI-NEB calculations at the DFT level constitute a separate validation stage. They are performed once on the final selected environments and do not feed back into the loop, so their error affects the confidence in the reported barrier reduction rather than the design.

The SAPT decomposition, the SOAP/UMAP analysis, and the SCScore distribution constitute post-hoc interpretation. They are applied to the final structures to characterize the interactions responsible for the observed stabilization, and are strictly downstream of both the optimization and the validation stages.

### Genetic algorithm

In the following, we describe how the GA sets up and optimizes the arrangement of molecules forming the local chemical environment of a transition state. The GA determines the type of molecule and its orientation/position.

#### Environment construction and parameterization

The first step in the genetic optimization phase is to construct a population of local chemical environments around a given reaction under investigation. In this work, we chose the environment to be five small organic molecules positioned along a set of predefined vectors, denoted by *S*, around a central reaction molecule (see Fig. 5a). This design is flexible; additional vectors can be added or removed to adjust the number of surrounding molecules as needed.

For each vector, *j*, a candidate molecule is selected from the SPICE dataset [52] (see Figure S1 in the SI for details on the data set), which was also used and re-computed to finetune the MACE-OFF23 model and train the initial generative model, and assigned to a vector. The position along the vector is denoted by $x_j$ and is typically chosen from a uniform distribution over a feasible range. Additionally, each molecule can be arbitrarily rotated. The rotation is parameterized by two angles, $\theta _j$ and $\phi _j$, which describe the orientation of the molecule within the rotation group SO(3). Including the molecular identifier i, the complete set of parameters for a molecule assigned to vector j is given by:

2 $$\begin{aligned} \left\{ (i, x_j, \theta _j, \phi _j) \;:\; j \in S \right\} . \end{aligned}$$

In our implementation, the use of five vectors yields five independent sets of parameters for each candidate environment. The placement of the five vectors around the transition state of the investigated reaction is shown in Fig. 5a.

#### Initial population and evaluation

An initial population of candidate environments is generated by repeating the above process n times. For each environment, a molecule is randomly selected for each vector j, and its corresponding parameters $(x_j, \theta _j, \phi _j)$ are generated randomly within their respective domains. This stochastic initialization ensures diversity in the initial search space.

The performance of each environment is then evaluated using property predictions with the finetuned MACE-OFF23 model in this work. The key metric is the interaction energy between the transition state and the environment, which is defined in Eq. (1).

#### Overlap phase (crossover)

Following evaluation, the GA proceeds by selecting the top-performing half of the population based on the defined score. These selected individuals form the basis for the crossover (or overlap) phase, which is designed to combine favorable traits from different environments (see Fig. 5c).

For each vector j, consider two parent environments with parameter sets:

3 $$\begin{aligned} \{(i^1, x_j^1, \theta _j^1, \phi _j^1), \quad (i^2, x_j^2, \theta _j^2, \phi _j^2)\}. \end{aligned}$$

A child environment is produced by randomly shuffling the parameter sets from the two parents. Specifically, for each parameter in the child’s configuration $C_j = \{(i^C, x_j^C, \theta _j^C, \phi _j^C)\}$, the value is chosen from the corresponding parameter of either parent with equal probability:

4 $$\begin{aligned} P(i^C = i^1) = P(i^C = i^2) = 0.5, \end{aligned}$$

and similarly for $x_j$, $\theta _j$, and $\phi _j$. This procedure is applied independently for every vector j, ensuring that the resulting child environment inherits a mix of characteristics from both parents. In the present work, this crossover operator was kept fixed and was not systematically varied. We chose this parameter-wise recombination because it provides a simple and unbiased mixing of parental configurations while remaining straightforward to implement. Although alternative crossover schemes could also be used, exploring them was beyond the scope of the present study. The crossover process is iterated until the population size is restored to its original number, thereby maintaining diversity while concentrating beneficial traits.

#### Mutation phase

To avoid premature convergence to local optima, a mutation step is incorporated into the GA (see Fig. 5d). During this phase, each parameter of the child environment is subjected to mutation with a fixed probability γ. The mutation introduces random changes, thereby allowing exploration of new regions in the parameter space.

Mathematically, for any given parameter:

5 $$\begin{aligned} P\bigl ((i^C, x_j^C, \theta _j^C, \phi _j^C) \rightarrow (i_M^C, x_j^C, \theta _j^C, \phi _j^C)\bigr ) = \gamma , \end{aligned}$$

6 $$\begin{aligned} P\bigl ((i^C, x_j^C, \theta _j^C, \phi _j^C) \rightarrow (i^C, x_j^C, \theta _j^C, \phi _j^C)\bigr ) = 1 - \gamma . \end{aligned}$$

Here, the mutation could affect the molecular index $i^C$, the position $x_j^C$, or the rotation angles $\theta _j^C$ and $\phi _j^C$. In this example the molecular index was effected by the mutation. New values for a mutated parameter are drawn from the same distributions used during the initial population generation. The mutation rate γ is chosen to balance the need for exploration (diversity) with the preservation of beneficial configurations. This mutation process is applied to every molecule in every candidate environment, forming the next generation of the population. The genetic optimization cycle, consisting of evaluation, crossover, and mutation, is repeated for 500 generations per hierarchical iteration. The outer hierarchical loop is then repeated until no further improvement in the best interaction energy is observed across iterations.

### Generative modeling

Once the GA has converged and a set of high-performing environments has been identified, the next phase involves generative modeling, i.e., molecular design. In this phase, the molecular configurations from the best-performing environments are collected and aggregated. Specifically, for each predefined vector j, the top-performing 5000 molecules (40 %) were aggregated by traversing the generations of the genetic algorithm starting from the last generation. This number was chosen to ensure a large enough chemical variability for the biasing step of the generative model [21]. The data sets, which capture the structural characteristics that contributed to a reduction in the activation energy, are combined to form a comprehensive dataset.

To generate the new set of candidate structures for each position j, the aggregated molecules from the previous iteration are used as reference data for a generative model. We used SiMGen [47], a diffusion-like generative model based on similarity kernels. SiMGen is a zero-shot local generator that moves atoms along the gradient of a time-dependent similarity score. In our runs, the descriptors $\{\boldsymbol{\chi }_i\}$ are the node representations extracted from the MACE-OFF23 model [53]. The similarity score is computed using a Gaussian radial basis function and is computed between all node descriptors in the reference $\{\boldsymbol{\chi }_i\}$ and generated structure $\{\boldsymbol{\chi }_j\}$. The kernel is defined as

7 $$\begin{aligned} k\!\left( \boldsymbol{\chi }_i,\boldsymbol{\chi }_j;t\right) = \exp \!\left[ -\,\frac{\bigl \Vert \boldsymbol{\chi }_i - \boldsymbol{\chi }_j \bigr \Vert ^2}{2\,\sigma (t)^2} \right] \, , \end{aligned}$$

where σ(t) is the kernel width dependent on time. Initially, this similarity kernel only includes an average restorative force that acts on the heavy atom backbone, guiding generated structures towards the average of the reference distribution. After this stage, hydrogen atoms are added in a separate refinement step and the resulting molecules are briefly relaxed using a machine-learned interatomic potential. All other parameters follow the public SiMGen defaults. The latent space in this work was provided by a pre-trained MACE-OFF23 model [49]. The use of this kernel-based generative model comes with the advantage that retraining of the generative model after each iteration is not required, only a set of reference structures has to be provided. By using the top-performing 40 % of subunits from the previous iteration to sample new subunits, the distribution of candidates is biased towards molecules with structural features more favorable to improve the target property.

To maintain a high quality of environment molecules, especially in later iterations, we applied filtering and relaxation steps (see section S2 in the SI for details). Therefore, the generated molecular geometries were optimized using MACE-OFF23 and molecules that did not converge were filtered out. Additional filtering criteria include charge neutrality, valid bond orders, and a minimum inter-heavy atom distance of 1.05 Å. As the optimization process relies on MACE-OFF23, finetuned on the SPICE data set (see Figure S2 for scatter plots indicating the model’s generally good performance) and not additionally finetuned on the designed molecules, a limitation of this approach could be that potential molecules might not converge due to high errors in forces. Still, due to the foundational knowledge underlying the model being trained on millions of organic molecules, this limitation is expected to concern only few molecular structures as a large range of chemical space is already covered. Additional active learning and sampling steps could reduce the risk of missing molecules due to potential holes in the fitted energy surface.

### MACE details

MACE models combine *E*(3)-equivariant message passing with an atomic–cluster–expansion (ACE) basis  [68–70]. The total energy is expressed as a sum of learned atomic contributions; when forces are included, they are computed by taking the derivative of the MACE model with respect to the atomic positions.

For energy predictions, the architectural parameters were taken from the MACE-OFF medium model [53]. A radial cutoff of $r_c=5~$Å was used to construct the local graph. The ACE includes explicit two and three body terms with angular channels up to $l_{\max }=3$. Distances were expanded using radial basis functions, specifically Bessel functions, angular components used spherical harmonics with coupling between them computed with the Clebsch Gordan coefficients, and atom types were encoded with learned embeddings. In order to finetune the energy prediction model a learning rate of $10^{-3}$ was used along with a learning decay of $5\cdot 10^{-10}$. The loss consists of the root mean squared errors of the energy. The number of epochs was set to 200. A scatter plot showing the performance of MACE finetuned on the SPICE data is shown in Figure S2 in the SI.

### SAPT(DFT) details

SAPT [59] was employed to decompose the intermolecular interaction energies between the transition state and the molecular subunits of the top-performing environments. SAPT is a perturbative method in which non-covalent interaction energies between two fragments are partitioned into physically meaningful components, namely electrostatics, exchange, induction and dispersion [59]. This allows for a more detailed interpretation of the physical interactions contributing to the stabilization of the transition state. In SAPT(DFT), the fragments are described by Kohn-Sham orbitals. The SAPT(DFT) calculations were carried out using Psi4 [71] at the PBE0 [60]/aug-cc-PVDZ level of theory. PBE0 was used in contrast to PBE for NEB calculations as only single point calculations were required and PBE0 is considered to be more accurate. For every of the 40 local chemical environments optimized with NEB (see sect. “NEB Details”), five SAPT(DFT) calculations were carried out. Each calculation involved two fragments: the transition state structure of the Claisen rearrangement and one of the five molecules constituting the environment. Splitting each environment into five pairwise SAPT(DFT) calculations allowed a more detailed decomposition of the interaction energies while reducing the overall computational cost.

### NEB details

NEB calculations were performed using the Atomic Simulation Environment (ASE) [72] interfaced with ORCA 6.0.0 [73] and xtb [74]. Prior to DFT (PBE/TZVP), we used GFN2-xTB for optimization. The NEB calculations were performed using climbing image. Due to the high computational cost of DFT, a convergence threshold of 0.15 eV/A was applied. Geometry optimizations employed the BFGS [75] algorithm with a convergence criterion of 0.15 eV/Å. DFT calculations were carried out with the PBE functional [76] in combination with the def2-TZVP basis set. To reduce the number of geometry optimization and NEB iterations, atoms in the surrounding environment were kept fixed.

## Supplementary Information

Supplementary material 1.

## Data Availability

All data and code generated for this work can be found at: https://doi.org/10.6084/m9.figshare.30265882.

## Code availability

The code for the generative model can be found at https://github.com/RokasEl/simgen. The associated MACE code and models for finetuning can be found at https://github.com/ACEsuit/mace.
