# Supplementary material for: Hierarchical generative modeling for the design of multi-component systems
Source: J Cheminform. 2026 Oct 11;18:140. doi: 10.1186/s13321-026-01313-3 (PMC13660158; doi:10.1186/s13321-026-01313-3)
Supplement: Supplementary file 1 [file 13321_2026_1313_MOESM1_ESM.pdf]

Supplementary Material for "Hierarchical generative modeling for the design of multi-component systems"

August 27, 2026

Contents

S1 Dataset construction and MACE-OFF23 finetuning **S2**

S2 MACE NEB Study **S3**

S2.1 Reference calculations S3

S2.2 Model finetuning S5

S3 Post-processing and molecular filtering **S5**

S4 Generative modeling **S7**

S5 Dimensionality reduction **S7**

S6 Claisen Rearrangement Reaction Lewis Structures **S9**

S7 Choice of SAPT(DFT) funcitonals **S9**

S8 Progress of Genetic Algorithm including Fifth Iteration **S9**

S9 Displacement of the transition state within the environment **S10**

S1 Dataset construction and MACE-OFF23 fine-tuning

The dataset for finetuning the medium MACE-OFF23 model [1], as well as for the first iteration of the GA, was constructed from the SPICE dataset [2]. The SPICE dataset provides an extensive set of small organic molecules, covering various chemical compositions and conformations. A filtering process was performed first using the following criteria, molecules were restricted to contain only elements implemented in the MACE-OFF models (H, C, N, O, F, P, S, Cl, Br, and I atoms), an even electron count and a size of 6–14 heavy atoms. Restricting the maximum number of heavy atoms to 14 ensures that during the genetic optimization cycles, the molecular sub-units collide less frequently during the assembly of valid populations. Distributions of size and atom types can be seen in Fig. S2. We define heavy atoms as any non-hydrogen atom.

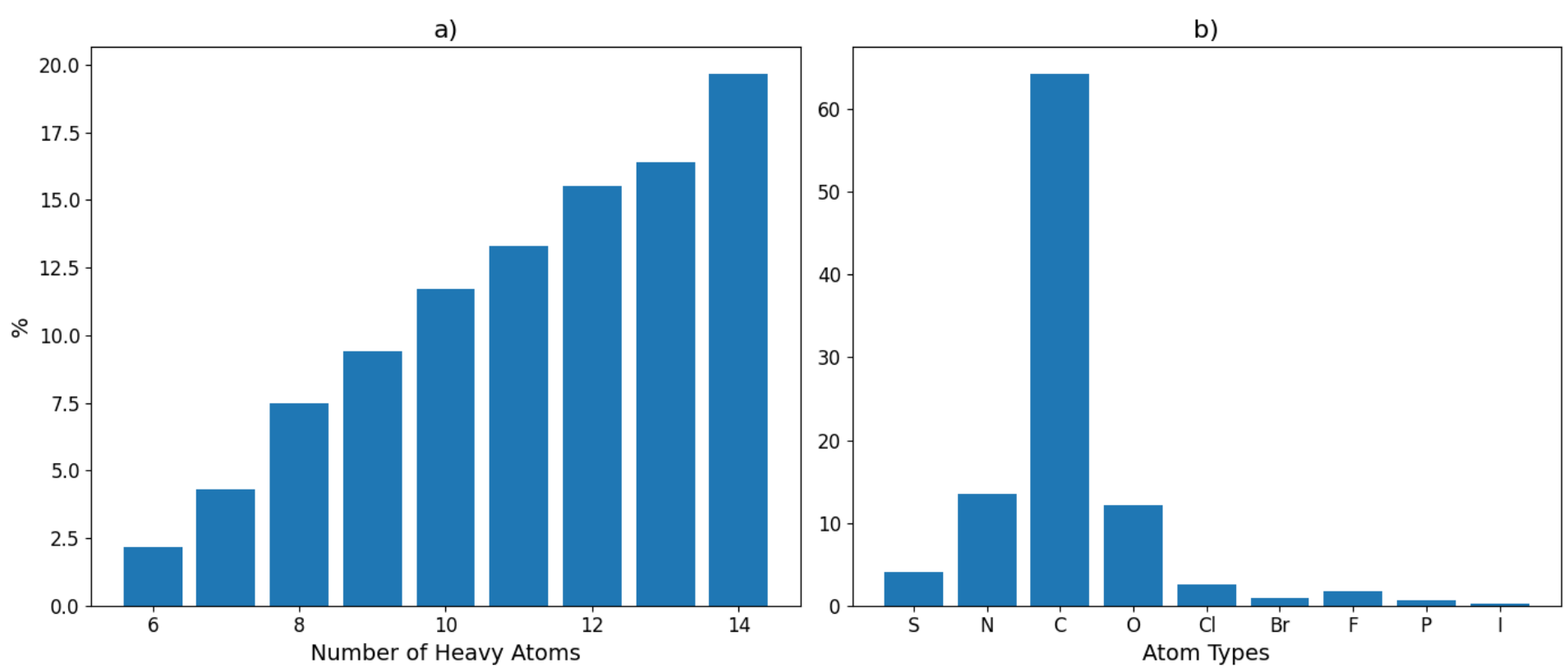

Figure S1: This figure shows the distribution of a) the number of heavy atoms and b) the atom type distribution of the initial molecular dataset obtained by sampling from the SPICE dataset.

To construct the initial dataset for training the supervised model, randomly selected molecules from selected SPICE molecules were combined with randomly generated orientations and distances. Distances were sampled between 2.5 and 3.5 Å, while orientations were uniformly sampled from 0 to 360 degrees.

S2 MACE NEB Study

Additionally, we compare transition-state geometries obtained from the MACE-OFF model NEB calculations and from ORCA NEB calculations for candidate environments selected in the final generative cycle. For each system, a corresponding image along the NEB pathway is extracted and the atomic structures are aligned using the Kabsch algorithm to remove overall translation and rotation. The root mean square deviation (RMSD) between atomic positions is then computed as a measure of structural similarity (Fig. S2a).

The resulting RMSD distribution is strongly peaked at low values, with the vast majority of structures exhibiting RMSDs below $\sim 0.2\,\text{Å}$ and a pronounced maximum in the first bin (≈ 0.01–$0.1\,\text{Å}$). This indicates that, for most candidates, the MACE-OFF model reproduces the DFT refined ORCA transition-state geometries with high fidelity. Only a small number of outliers extend to larger deviations (up to ~ 1.5–$1.8\,\text{Å}$), corresponding to cases where the ML model and ORCA optimization diverge more significantly. Overall, the distribution demonstrates that MACE-OFF captures the local transition-state geometry accurately across the final candidate set, while highlighting that accurate structural agreement does not necessarily translate directly into quantitative agreement in activation energies.

To further assess this, we compute the differences in activation energies between the two methods for each system, defined as the difference in barrier heights ($\Delta\Delta E$) extracted from the corresponding NEB energy profiles (Fig. S2b). This provides a direct measure of agreement in the predicted energetics across all images.

Despite this strong structural agreement, the activation energies predicted by MACE-OFF can deviate from those obtained with ORCA. This is likely due to differences in the underlying levels of theory, as well as the fact that the present system may not be well represented in the training distribution of the MACE-OFF model. Fine tuning the model, as discussed in the original work, can help alleviate these discrepancies.

S2.1 Reference calculations

To reduce the computational cost of dataset generation, interaction energies were computed from pairwise DFT calculations between the reaction transition state and each molecule in the environment. Calculations were performed at the range-separated hybrid functional wB97M-D3BJ [3] level, which incorporates Grimme's D3BJ dispersion correction [4] to account for long-range interactions, in combination with the def2-TZVP basis set, a triple

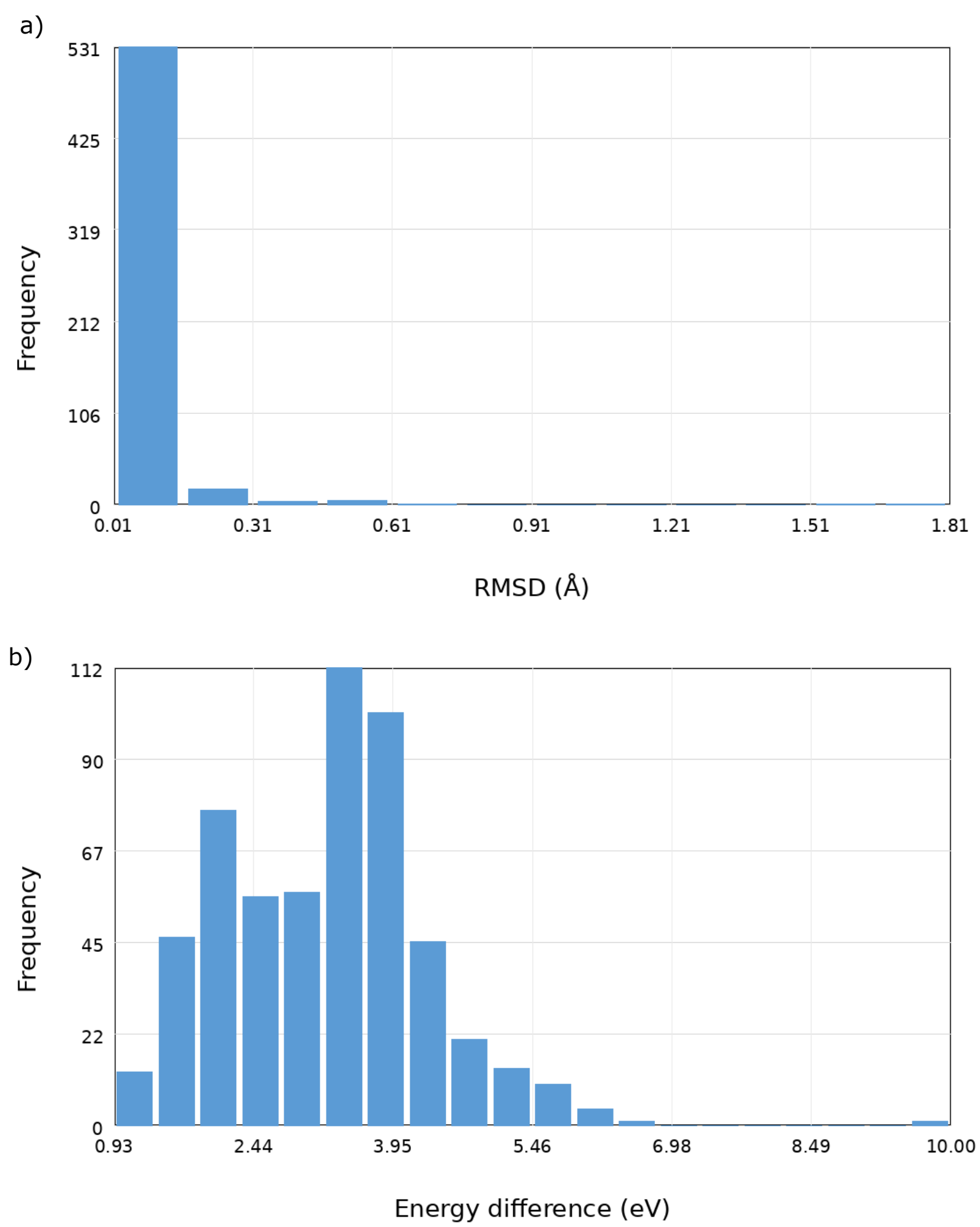

Figure S2: (a) Histogram of RMSDs between transition-state region structures obtained from MACE-OFF and ORCA NEB calculations for candidate environments from the final generative cycle. For each system, a corresponding image along the NEB pathway is extracted and the structures are aligned using the Kabsch algorithm prior to RMSD evaluation. (b) Histogram of energy differences between the two methods across all images in the NEB.

zeta valence basis with polarization functions.

For each pair, the total energy of the combined system (transition state plus a single environment molecule) was calculated, together with the energies of the two individual molecules. The interaction energy was defined as the difference between the combined energy and the sum of the two monomer energies. Finally, the total interaction energy of the environment was obtained by summing all pairwise contributions across the set of environmental molecules.

S2.2 Model finetuning

The MACE-OFF23 medium model was used as the starting point for finetuning on the dataset of 36,667 reference calculations using the method defined above. The training was performed for 200 epochs with a learning rate of 10^{-3}. The final model was evaluated on the test set by predicting the energies and comparing to the reference values from the DFT calculations. The corresponding scatterplot is shown in Fig. S3.

S3 Post-processing and molecular filtering

To ensure that the molecules generated by the SiMGen model were both chemically valid and physically meaningful, molecules were filtered based on predefined criteria. As a first step, all candidate structures were subjected to geometry optimization using the MACE-OFF model. Only those molecules that could be optimized were retained. This criterion ensured that molecules correspond to genuine stationary points on the potential energy surface rather than unstable configurations. In addition, each structure was sorted based on the number of electrons, such that only molecules with an even number of electrons were retained, ensuring closed-shell states and excluding radicals or other open-shell species. Structural plausibility was further enforced by having minimum interatomic distance thresholds, preventing the inclusion of molecules with unrealistically short bond lengths or atomic clashes. All remaining structures were required to be convertible into valid molecular graphs using RDKit[5] with consistent bond orders and chemically valid connectivity.

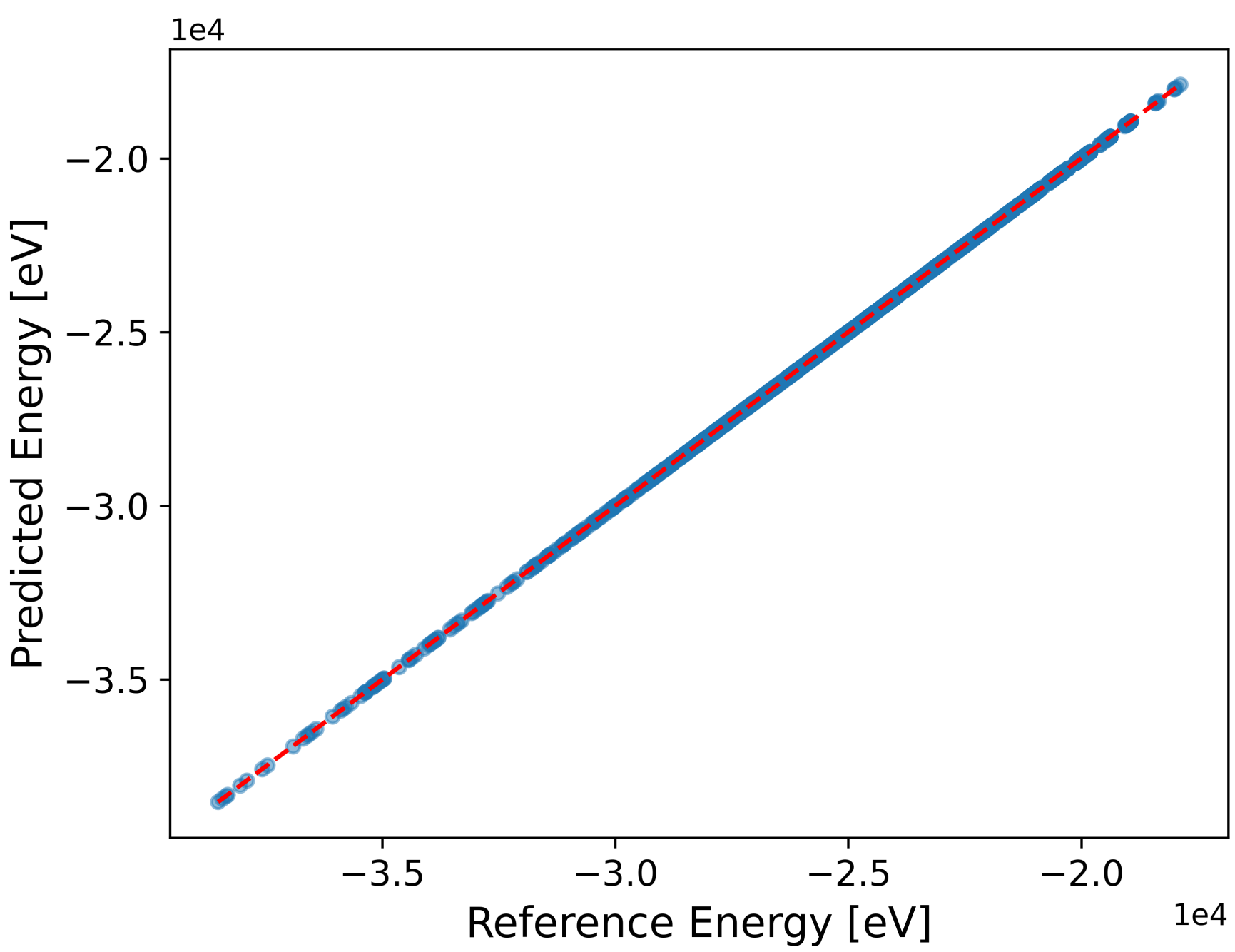

Figure S3: Reference energies of the test set versus predicted energies of the finetuned MACE-OFF23 medium model.

S4 Generative modeling

To further validate generated molecules with SiMGen [6], bond angles and bond lengths were calculated and compared across successive iterations, with the first iteration constructed from the SPICE dataset. We have selected C-C, C-N, and C-O bond lengths as well as corresponding angles as examples to compare distributions. The distributions of the selected bond-lengths and angles can be seen in S4. In all cases, distributions remained closely aligned with those of the original dataset, indicating that the generated molecules preserve structural characteristics.

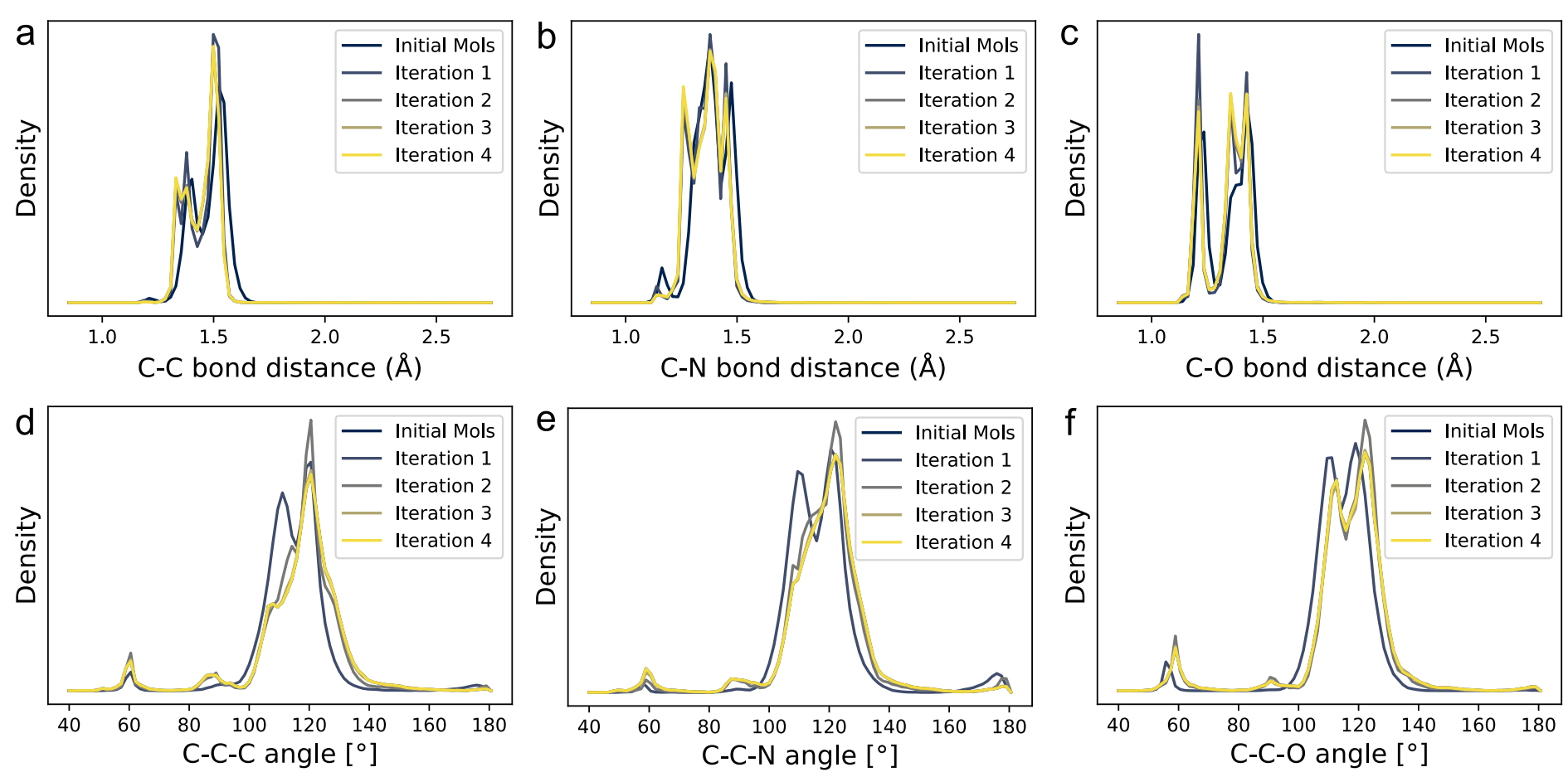

Figure S4: Distributions of a) C-C, b) C-N, and c) C-O bonds as well as d) C-C-C, e) C-C-N, and f) C-C-O bond angles.

S5 Dimensionality reduction

To analyze how the distribution of generated molecules evolves across chemical space, the Smooth Overlap of Atomic Positions (SOAP) descriptor [7] was calculated for the initial molecular dataset and for the datasets of extracted sub-unit molecules after each iteration of the workflow. SOAP represents atomic environments by expanding neighbor densities into radial and spherical basis functions and comparing them via smooth overlaps, yielding rotation- and permutation-invariant descriptors for molecular geometries. We used four radial basis functions, a maximum spherical harmonics angular momentum of three and a cutoff radius of three Å. Due to variable

numbers of atoms per molecule, we used inner-product aggregation of the atomic descriptors to obtain consistent descriptor-shapes for each molecule descriptor. For the computation of the soap descriptors, we used the dscribe python package [8]. To visualize the high-dimensional descriptor space in a lower-dimensional space, we performed Uniform Manifold Approximation and Projection (UMAP) [9] dimensionality reduction. UMAP works by constructing a weighted graph of local neighborhoods to approximate the manifold structure of high-dimensional data and then optimizing a low-dimensional embedding that preserves those neighborhood relationships. For the UMAP projection of the SOAP descriptors, we used the umap python package [10]. The number of neighbors was set to 50, the minimum distance parameter was set to 0.1 and we chose to use the cosine similarity as a distance metric. For the UMAP projection plot in Figure 2c of the main paper, performed a joint dimensionality reduction of all SOAP descriptors and colored the points by iteration of the workflow. The UMAP projection of each individually, colored by workflow iteration is shown in Figure S5.

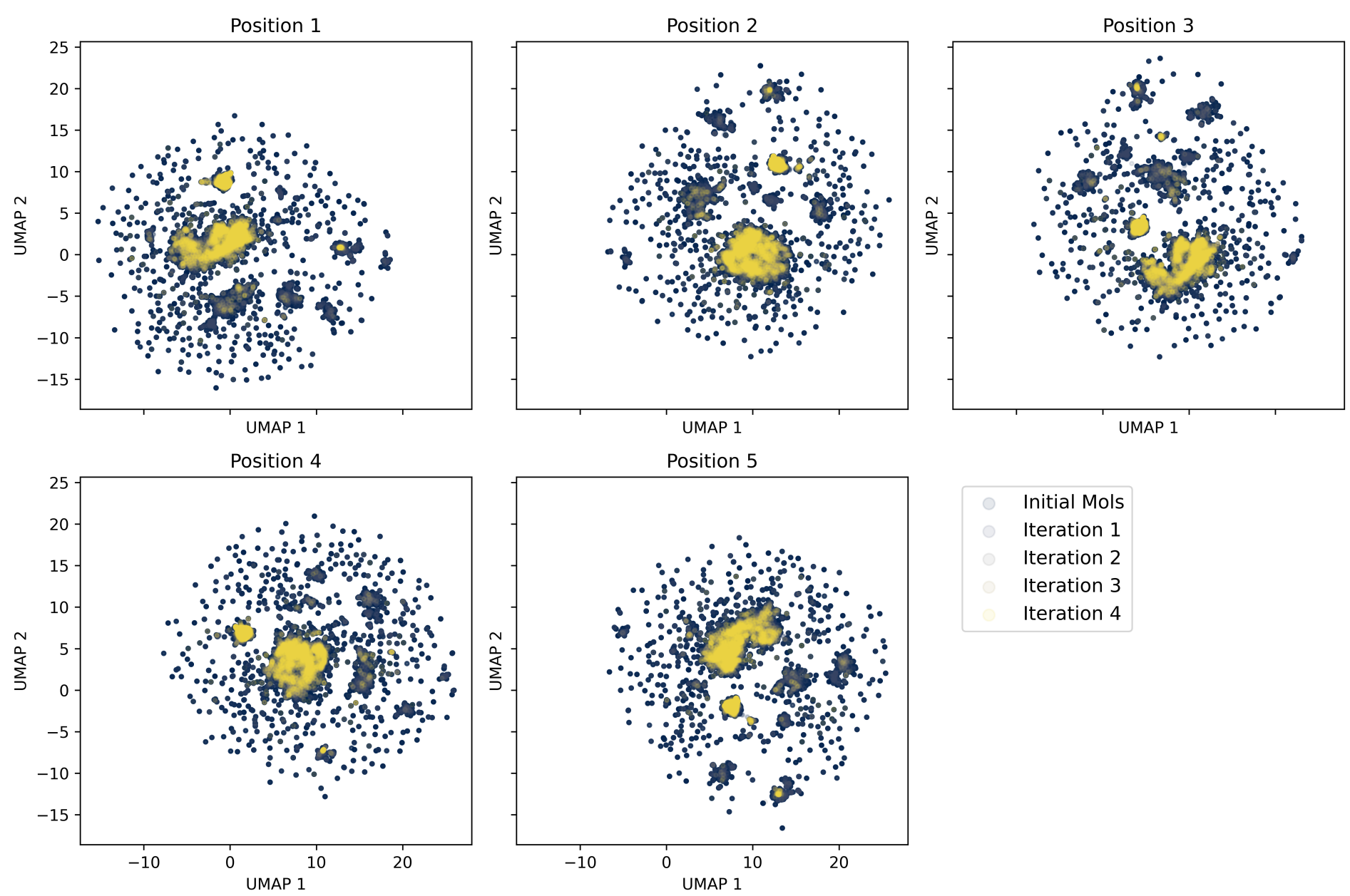

Figure S5: UMAP projections of the initial molecule dataset sampled from SPICE and the datasets generated during the different iterations of our workflow for each of the five local positions.

S6 Claisen Rearrangement Reaction Lewis Structures

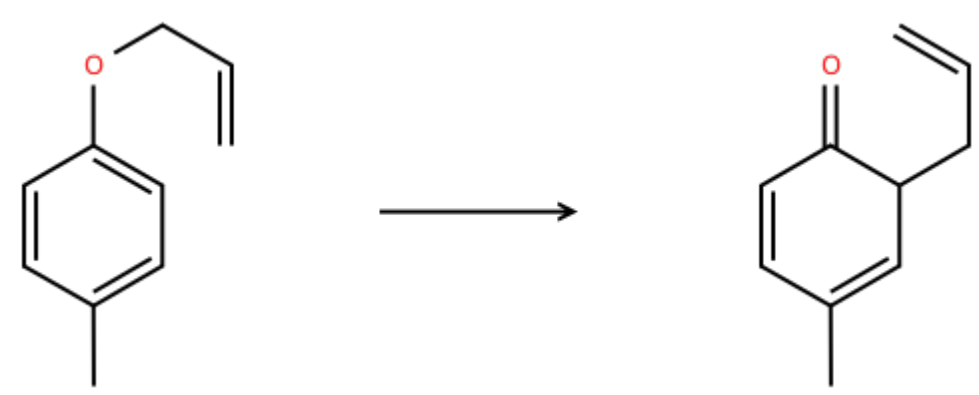

Figure S6: Lewis structures of reactant and product structures in the studied reaction

S7 Choice of SAPT(DFT) funcitonals

The SAPT(DFT) calculations were carried out using PSI4 [11]. While the reference interaction-energy calculations used wB97M, this meta-GGA functional is not supported in the PSI4 SAPT(DFT) implementation. PBE0 [12] was therefore used as a supported hybrid functional for SAPT(DFT). The aug-cc-pVDZ basis was selected because augmented correlation-consistent basis sets include diffuse functions, which are important for accurately describing long-range polarization effects and the induction and dispersion contributions in noncovalent interactions." PBE0 was used in contrast to PBE for NEB calculations as only single point calculations were required and PBE0 is considered to be more accurate.

S8 Progress of Genetic Algorithm including Fifth Iteration

To further assess convergence of the outer hierarchical loop, we performed an additional fifth iteration consisting of another full GA optimization over 500 generations followed by generative updating of the subunit pool (Fig. S7). As shown, the fifth iteration did not improve the mean interaction energy beyond the best values obtained in iterations 3 and 4, but instead converged to a higher final value. We therefore stopped the iterative procedure after four reported iterations. In addition, later iterations showed a growing fraction of unstable or invalid candidate structures, further reducing the effective yield of useful environments.

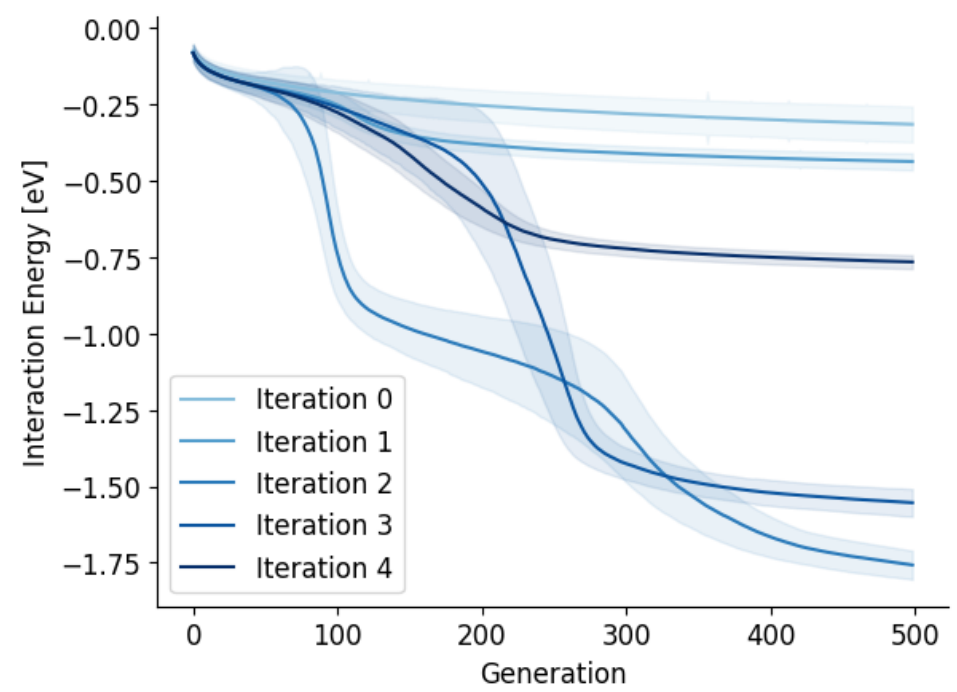

Figure S7: Progress of the genetic algorithms. Solid lines represent the mean interaction energy of all generated local chemical environments with respect to the reference transition state and shaded areas show the standard deviation.

S9 Displacement of the transition state within the environment

The interaction energy used inside the genetic algorithm is evaluated with respect to a fixed gas-phase transition-state geometry, so the search itself does not allow the transition state to relax in response to the surrounding environment. The barriers reported in the main text are instead obtained from climbing-image NEB calculations in which the reacting complex is relaxed within the environment, with the environment molecules held fixed to limit computational cost. To quantify how far the transition state moves once this relaxation is permitted, we compare the gas-phase reference geometry with the climbing-image geometry obtained from CI-NEB in the presence of the environment.

For each system, the 23 atoms of the reacting complex are extracted from the environment-containing pathway and aligned to the gas-phase reference using the Kabsch algorithm to remove overall translation and rotation. The image at the top of the barrier is identified from the NEB energy profile rather than by a fixed image index. We then report both the root mean square deviation (RMSD) over the reacting complex and the largest displacement of any single atom.

Across the 40 environments for which a CI-NEB calculation was completed, the RMSD distribution is unimodal and concentrated at low values, with a mean of 0.26 Å and a median of 0.24 Å (Fig. S8a). Displacements are small in

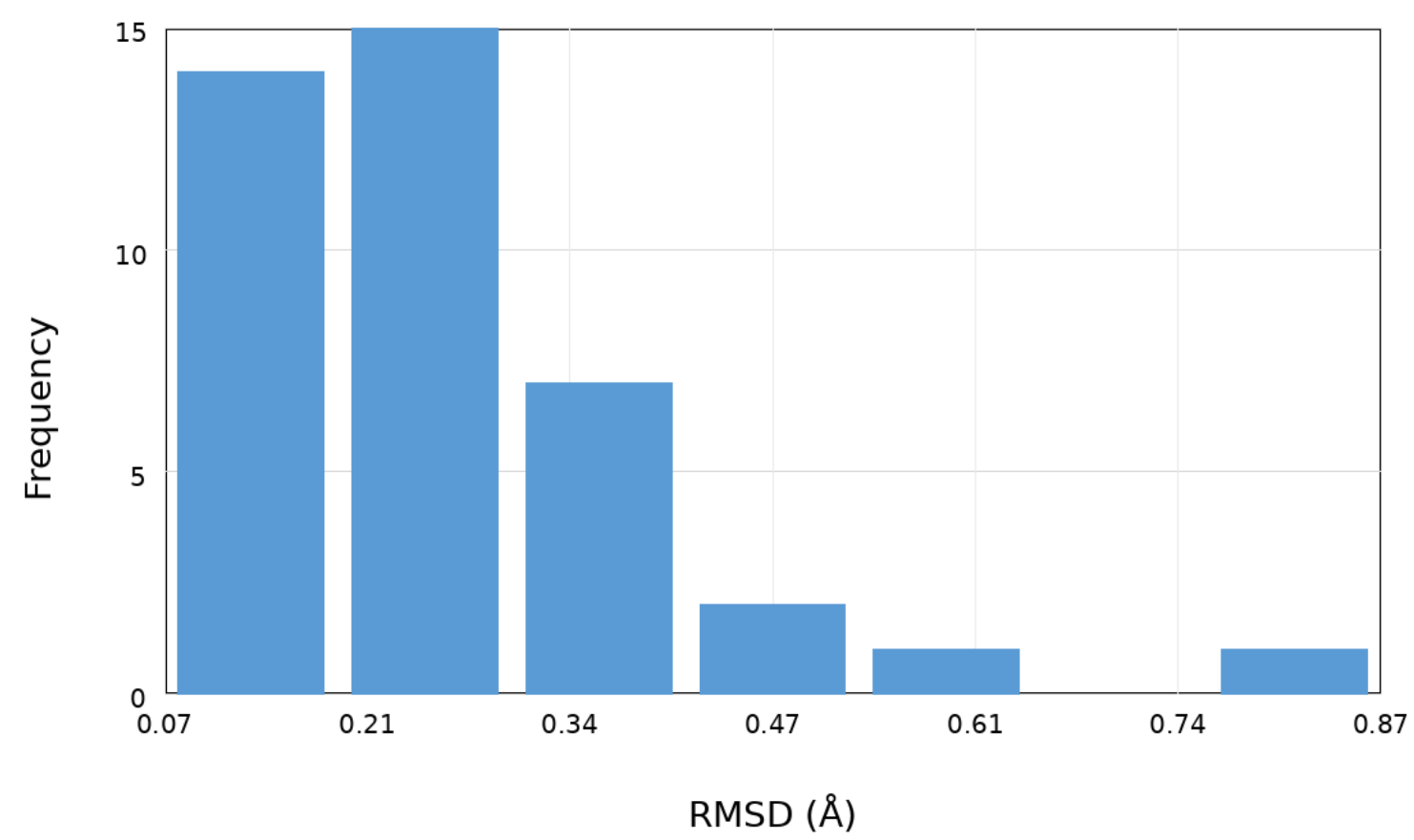

Figure S8: Displacement of the transition state upon relaxation within the local chemical environment. (a) Histogram of RMSDs between the gas-phase reference transition state and the climbing-image geometry obtained from CI-NEB within the environment, for the 40 environments with a completed NEB calculation.

the large majority of cases: 29 of 40 environments lie below 0.3 Å and 35 of 40 below 0.4 Å, with a 90th percentile of 0.41 Å. One environment is a clear outlier at 0.87 Å; the next largest deviation is 0.59 Å. The corresponding maximum single-atom displacements are larger, as expected relative to a whole-structure measure, with a median of 0.45 Å and a 90th percentile of 0.73 Å. Apart from the single outlier, we observe no distinct second population at larger deviations, indicating that the transition state shifts modestly in essentially all environments rather than remaining static in most and reorganising substantially in a few.

Fig. S9 shows what a deviation of this size corresponds to structurally, for the environment lying closest to the mean of the distribution. The deviation is dominated by hydrogen: the eleven heavy atoms give an RMSD of 0.20 Å against 0.30 Å for the twelve hydrogens, and the three largest single-atom displacements all belong to the methyl group, whose torsion changes by approximately 6°. More importantly, the partial bonds defining the saddle point are preserved, the breaking C-O distance changes from 2.10 to 2.20 Å and the forming C-C distance from 2.14 to 2.29 Å. The environment therefore shifts the transition state marginally earlier along the reaction coordinate without altering its character.

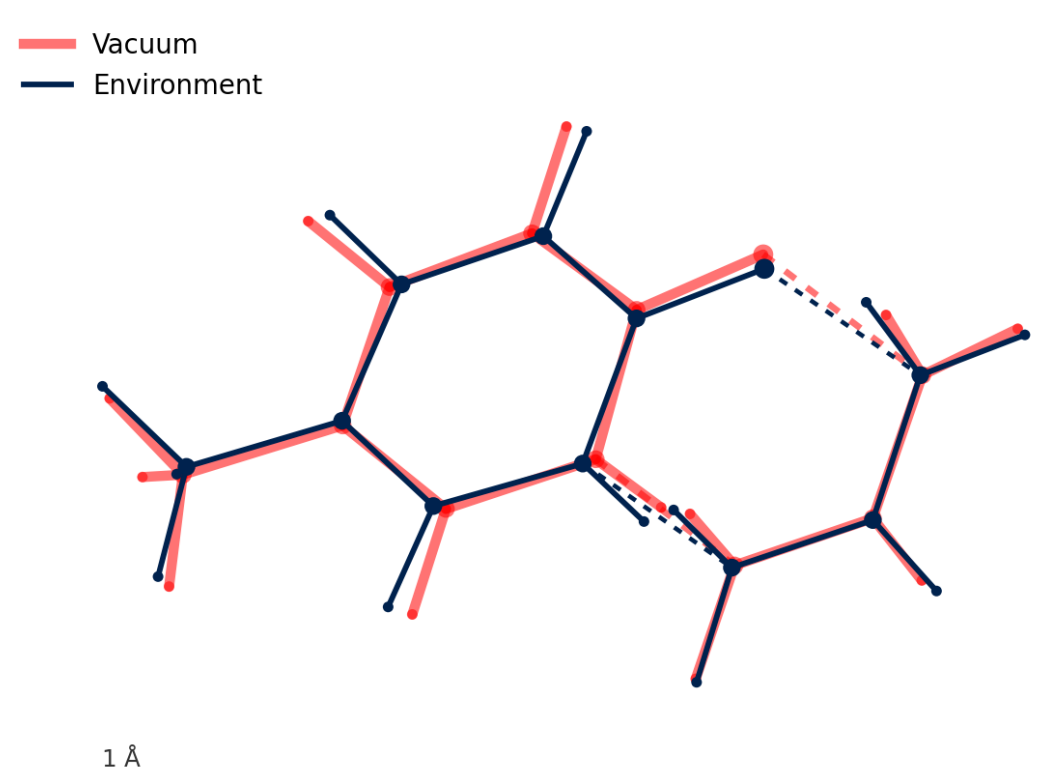

Figure S9: Gas-phase reference transition state (red) overlaid with the climbing-image geometry relaxed within the environment (navy), for the environment closest to the mean RMSD (0.26 Å).

References

(1) Kovács, D. P.; Moore, J. H.; Browning, N. J.; Batatia, I.; Horton, J. T.; Pu, Y.; Kapil, V.; Witt, W. C.; Magdau, I.-B.; Cole, D. J., et al. *J. Am. Chem. Soc.* **2025**, *147*, 17598–17611.

(2) Eastman, P.; Behara, P. K.; Dotson, D. L.; Galvelis, R.; Herr, J. E.; Horton, J. T.; Mao, Y.; Chodera, J. D.; Pritchard, B. P.; Wang, Y.; De Fabritiis, G.; Markland, T. E. *Sci. Data* **2023**, *10*, 11.

(3) Mardirossian, N.; Head-Gordon, M. *J. Chem. Phys.* **2016**, *144*.

(4) Grimme, S.; Ehrlich, S.; Goerigk, L. *J. Comput. Chem.* **2011**, *32*, 1456–1465.

(5) Landrum, G. *Release* **2013**, *1*, 4.

(6) Elijošius, R.; Zills, F.; Batatia, I.; Norwood, S. W.; Kovács, D. P.; Holm, C.; Csányi, G. *Nat. Commun.* **2025**, *16*, 5991.

(7) Bartók, A. P.; Kondor, R.; Csányi, G. *Phys. Rev. B* **2013**, *87*, 184115.

(8) Himanen, L.; Jäger, M. O. J.; Morooka, E. V.; Federici Canova, F.; Ranawat, Y. S.; Gao, D. Z.; Rinke, P.; Foster, A. S. *Comput. Phys. Commun.* **2020**, *247*, 106949.

(9) McInnes, L.; Healy, J.; Melville, J. *arXiv* **2018**.

(10) McInnes, L.; Healy, J.; Saul, N.; Grossberger, L. *J. Open Source Softw.* **2018**, *3*, 861.

(11) Parrish, R. M.; Burns, L. A.; Smith, D. G.; Simmonett, A. C.; DePrince III, A. E.; Hohenstein, E. G.; Bozkaya, U.; Sokolov, A. Y.; Di Remigio, R.; Richard, R. M., et al. *J. Chem. Theory Comput.* **2017**, *13*, 3185–3197.

(12) Adamo, C.; Barone, V. *J. Chem. Phys.* **1999**, *110*, 6158–6170.
